# Characterization of multi-stage metabolic alterations in hepatitis B virus-related acute-on-chronic liver failure using high-coverage metabolomics

**DOI:** 10.1007/s11306-026-02462-0

**Published:** 2026-06-16

**Authors:** Zhehua Zhang, He Jiang, Guoqi Zhang, Deying Chen, Xiaoling Su, Liang Li, Lanjuan Li

**Affiliations:** 1 State Key Laboratory for Diagnosis and Treatment of Infectious Diseases, China-Singapore Belt and Road Joint Laboratory on Infection Research and Drug Development, National Clinical Research Center for Infectious Diseases, National Medical Center for Infectious Diseases, Collaborative Innovation Center for Diagnosis and Treatment of Infectious Diseases, The First Affiliated Hospital, Zhejiang University School of Medicine, Hangzhou, China; 2 Yuhang Institute of Medical Science Innovation and Transformation, Hangzhou, China; 3 Department of Critical Care Medicine, Shulan (Hangzhou) Hospital Affiliated to Zhejiang Shuren University Shulan International Medical College, Hangzhou, China; 4 The Metabolomics Innovation Centre and Department of Chemistry, University of Alberta, Edmonton, AB Canada

**Keywords:** Metabolomics, Chemical isotope labeling, HBV-ACLF, COSSH

## Abstract

**Introduction:**

Hepatitis B virus-related acute-on-chronic liver failure (HBV-ACLF) is a serious condition characterized by acute liver decompensation and multiple organ failure, which leads to high short-term mortality rates.

**Objectives:**

This study aims to understand the metabolic changes across different stages—normal, chronic hepatitis B (CHB), and HBV-ACLF—to provide important insights into disease progression and potential diagnostic markers.

**Methods:**

This retrospective study analyzed serum samples from 125 HBV-ACLF patients (49 at ACLF-1, 47 at ACLF-2, and 29 at ACLF-3) classified according to the COSSH criteria, along with 34 patients with CHB and 32 healthy controls (HCs). High-sensitivity untargeted metabolomic profiling was performed using chemical isotope labeling (CIL) techniques.

**Results:**

A total of 2053 amine/phenol-containing metabolites were detected. Significant metabolic alterations were identified, including 32 positively identified metabolites exhibiting persistent changes initiating at the CHB stage. This number increased to 91 in ACLF-1, decreased to 51 in ACLF-2, and further decreased to 38 in ACLF-3. Specifically, we observed increased levels of γ-glutamyl dipeptides and metabolites from the tryptophan-kynurenine pathway, while levels of metabolites from the tryptophan-serotonin pathway decreased. Additionally, metabolic pathways involving cysteine, methionine, and taurine displayed activation, indicating a compensatory response to oxidative stress and liver dysfunction.

**Conclusion:**

This study identifies distinct metabolic trajectories from normal liver function to CHB and ultimately to HBV-ACLF, contributing to understand the relationship between liver dysfunction and systemic inflammation. Our findings may facilitate the development of diagnostic biomarkers and tailored therapeutic strategies. Further research is needed to explore the mechanisms behind these metabolic changes and their relevance to managing patients with HBV-ACLF.

**Supplementary Information:**

The online version contains supplementary material available at https://doi.org/10.1007/s11306-026-02462-0.

## Introduction

Acute-on-chronic liver failure (ACLF) is a life-threatening syndrome occurring in patients with chronic liver disease, characterized by acute liver decompensation and single- or multiple-organ failure, resulting in a high 28 day mortality rate (Li et al., 2023; Moreau et al., 2013). Hepatitis B virus-related ACLF (HBV-ACLF) is the predominant form of ACLF in the Asia–Pacific region, primarily triggered by acute hepatic insults from the reactivation of HBV in individuals with chronic hepatitis B (CHB) (Sarin et al., 2019). A large prospective multicenter study conducted by the Chinese Group on the Study of Severe Hepatitis B (COSSH) demonstrated that HBV-ACLF presents distinct clinical characteristics, including a higher incidence of liver failure and coagulation failure, as well as significantly elevated short-term mortality compared to non-HBV-ACLF cases (Wu et al., 2018a).

The syndrome of HBV-ACLF is marked by rapid changes in its clinical trajectory, with most patients progressing to their final ACLF grade within 3 to 7 days of diagnosis (Zhang et al., 2021). Besides, the underlying clinical conditions of HBV-ACLF patients could further complicated the disease progression and impacts prognosis (Chen et al., 2025). Consequently, in-depth investigations into the pathophysiological mechanisms underlying the swift progression of HBV-ACLF are essential for making timely therapeutic decisions, delaying disease progression, and improving patient outcomes.

Metabolomics, which closely links to phenotype expression, can rapidly and accurately reflect dynamic pathological changes (Guijas et al., 2018; Qiu et al., 2023). Recent metabolomics studies have shown dramatic alterations in metabolic profiles during ACLF, including intense peripheral catabolic metabolism, suppressed mitochondrial β-oxidation and oxidative phosphorylation (OXPHOS), impaired membrane lipid biosynthesis, and remodeled amino acid metabolism (Moreau et al., 2020; Zhang et al., 2023, 2024a, 2024b). Moreover, ACLF altered the distribution characteristics of the gut bacterial groups (Ye et al., 2025). Elevated serum levels of gut microbiome-derived metabolites, such as aromatic compounds, bile acids, benzoate, and estrogen metabolites, have been associated with poor outcomes in ACLF patients (Bajaj et al., 2020). However, stage-specific metabolic perturbations throughout HBV-ACLF progression have yet to be adequately explored.

Low‑abundance polar metabolites (amino acids, biogenic amines, indoles and phenolics) are key products of host amino acid metabolism and gut microbial metabolism (Oliphant & Allen-Vercoe, 2019). While exerting essential functions in hepatic metabolism and gut–liver signaling (Hu et al., 2025), these metabolites remain underrepresented in conventional untargeted workflows, owing to pronounced ion suppression in complex biological matrices and the limited separation capability for highly hydrophilic compounds (Vuckovic, 2018). Meanwhile, chemical isotope labeling (CIL) LC–MS significantly enhanced both ionization efficiency and chromatographic retention of analytes through rationally designed differential isotope tags (Zhao et al., 2019). The differential ^12^C-/^13^C-isotope dansylation labeling LC–MS has proven effective for characterizing and quantitatively analyzing the amine/phenol submetabolome, revealing distinct advantages in elucidating disease mechanisms and assessing therapeutic efficacy (Chen et al., 2024; Sun et al., 2018; Wang et al., 2024).

In this study, we enrolled normal controls, CHB patients, and ACLF patients with different stages according to COSSH criteria. Utilizing ^12^C-/^13^C-dansylation labeling LC–MS, we profiled and quantified the metabolic dysregulations associated with HBV-ACLF. This comparative design enables a clearer characterization of specific metabolomic signatures associated with disease progression and highlight the critical metabolic shifts involved. Our findings may pave the way for more effective diagnostic and therapeutic approaches.

## Materials and methods

### Patients

This retrospective study enrolled 34 patients with CHB, 125 patients with HBV-ACLF and 32 healthy controls (HCs) at the First Affiliated Hospital, Zhejiang University School of Medicine in Hangzhou, China, between January 2017 and December 2023. The healthy controls were recruited from the Health Management Center. CHB patients were diagnosed based on persistent HBsAg positivity, elevated liver transaminases, and liver biopsy‑proven hepatic inflammation. HBV-ACLF patients were diagnosed according to the guidelines of COSSH-ACLF, and were divided into three subgroups based on the frequency of organ failures, namely, ACLF-1, ACLF-2, and ACLF-3 (Wu et al., 2018a). Detailed inclusion criteria for HCs, CHB, and HBV-ACLF, as well as exclusion criteria for HBV-ACLF, were provided in Supplementary Materials (Supplemental Note S1).

The study protocol was approved by the Ethics Committee of the First Affiliated Hospital, Zhejiang University School of Medicine (Approval number: 2024-1331, Date of approval: November 26, 2024), and informed consent was obtained from all participants.

### Clinical information collection

The general clinical characteristics of the study participants were retrieved from outpatient medical records. Clinical information encompassed demographic information, hepatic and renal function parameters, HBV infection markers, hematological and coagulation profiles, lipid and energy metabolism indicators, electrolyte levels, as well as biomarkers for hepatocyte regeneration and necrosis. The complete list of all collected clinical parameters was detailed in Supplementary Materials (Supplemental Note S1).

### Metabolomics analyses

Serum samples were prepared from fasting venous blood of participants and stored at − 80 °C until analysis. Prior to dansylation labeling, all the serum samples were thawed on ice, then mixed with three volumes of pre‑chilled methanol for complete protein precipitation, and subsequently vacuum‑dried using a SpeedVac vacuum concentrator (Labconco, USA). A 10 μL aliquot of serum from each individual sample was combined to create a pooled sample, which was processed using the same protein precipitation procedure.

Dansylation was carried out according to an established protocol (Wang et al., 2024). Individual samples were labeled with ^12^C-dansyl chloride. The pooled sample was labeled with ^13^C-dansyl chloride to serve as a reference for relative quantification. An equal volume of ^12^C-dansyl labeled individual sample was mixed with ^13^C-dansyl labeled pooled sample before LC–MS analysis. Quality control (QC) samples were prepared by mixing equal volume of ^12^C- and ^13^C-labeled pooled samples.

All samples were randomised for LC–MS analysis using Ultimate 3000 UHPLC (Thermo Scientific, USA) coupled with Impact II Q-TOF (Bruker, USA) under established conditions with minor modifications (Zhang et al., 2022).

Detailed information of sample collection and preparation, dansylation labeling and LC‑MS parameters were presented in Supplemental Note S2 of the Supplementary Materials.

### Data processing and statistical analysis

Peak pairs of ^12^C/^13^C-dansyl labeled metabolites were extracted, aligned, and subjected to missing value retrieval using IsoMS Pro (Zhou et al., 2014). Metabolite identification was conducted employing a previously established three-tiered strategy (Zhao et al., 2019).

Principal component analysis (PCA) and partial least squares discriminant analysis (PLS-DA) were performed using the software SIMCA-P 14.1 (Umetrics AB, Sweden) and MetaboAnalyst 6.0 for comprehensive data interpretation. Venn diagram was produced by jvenn (Bardou et al., 2014). Statistical analyses were conducted utilizing IBM SPSS 22 (IBM, USA). The normality of continuous variables was assessed using the Shapiro–Wilk test. Normally distributed continuous variables were expressed as mean ± standard deviation, while non-normally distributed variables were presented as median (interquartile range). Categorical data were expressed as percentages. For continuous variables, binary comparisons were performed with the Student’s t-test (normal distribution) or Mann–Whitney U test (non-normal distribution), while comparisons across multiple groups were analyzed using one-way ANOVA (normally distributed) or the Kruskal–Wallis test (non-normally distributed). Categorical variables were compared using the chi-square test or Fisher’s exact test. All pairwise post-hoc comparisons were performed only when the corresponding omnibus test showed a statistically significant difference. Bonferroni-corrected Student’s t-tests, Dunn’s test with Bonferroni correction, and Bonferroni-corrected pairwise chi-square partition were used for post-hoc comparisons following one-way ANOVA, Kruskal–Wallis test, and chi-square test, respectively.

## Results

### Demographic and clinical information

The clinical characteristics of CHB patients, HBV-ACLF patients, and healthy controls are summarized in Table 1. The analysis revealed no significant differences in age distribution among HCs, CHB patients, and HBV-ACLF patients. However, a clear observation was the significantly higher proportion of male patients in the HBV-ACLF group compared to both the HCs and CHB groups, suggesting a gender-related predisposition to severe disease.

**Table 1:** Patient characteristics at enrollment

Characteristics	HC (n = 32)	CHB (n = 34)	HBV-ACLF (n = 125)	*p*^a^ Value	*p*^b^ Value	*p*^c^ Value	*p*^d^ Value
Age (years)	45 ± 9	43 ± 10	47 (37–55)	0.391	NA	NA	NA
Male (%)	15 (46.9%)	22 (64.7%)	107 (85.6%)	< 0.001	0.434	< 0.001	0.017
ALB (g/L)	44.9 ± 1.8	41.2 ± 5.1	30.8 (28.7–32.7)	< 0.001	0.494	< 0.001	< 0.001
ALT (U/L)	16 (11–20)	281 (136–562)	270 (128–511)	< 0.001	< 0.001	< 0.001	1.000
AST (U/L)	18 ± 3	149 (72–216)	174 (106–291)	< 0.001	< 0.001	< 0.001	0.551
ALP (U/L)	72 ± 18	115 ± 29	143 (123–175)	< 0.001	< 0.001	< 0.001	< 0.001
ChE (U/L)	8105 ± 1345	7213 ± 2125	3766 (2972–4891)	< 0.001	0.426	< 0.001	< 0.001
TBA (μmol/L)	4.0 ± 1.6	13.2 (5.7–38.1)	304.8 (210.0–427.8)	< 0.001	0.093	< 0.001	< 0.001
TBil (μmol/L)	10.3 (8.7–13.5)	17.2 (11.1–25.2)	363.1 (305.4–457.7)	< 0.001	0.476	< 0.001	< 0.001
DBil (μmol/L)	4.0 (3.6–4.7)	8.2 (5.4–17.9)	256.6 (218.9–362.6)	< 0.001	0.196	< 0.001	< 0.001
IBil (μmol/L)	6.1 (4.7–8.7)	7.5 (5.9–11.5)	81.7 (61.1–115.8)	< 0.001	1.000	< 0.001	< 0.001
ADA (U/L)	9.0 (8.0–10.0)	20.2 (15.3–29.0)	29.7 (25.0–35.2)	< 0.001	< 0.001	< 0.001	0.001
GGT (U/L)	15 (11–25)	133 (55–210)	75 (49–102)	< 0.001	< 0.001	< 0.001	< 0.001
GFR (mL/min)	99.8 ± 12.0	104.7 (97.6–111.9)	109.4 (97.0–120.5)	0.020	1.000	0.027	0.387
Cr (μmol/L)	71 ± 14	75 (62–79)	63 (55–75)	0.007	1.000	0.130	0.017
BUN (mmol/L)	4.85 ± 1.06	3.99 (3.14–5.11)	3.99 (2.81–5.74)	0.099	NA	NA	NA
TG (mmol/L)	1.00 ± 0.31	1.27 (0.86–1.62)	1.02 (0.88–1.29)	0.048	0.041	0.334	0.392
TC (mmol/L)	4.36 ± 0.69	4.24 ± 0.95	1.88 (1.52–2.39)	< 0.001	1.000	< 0.001	< 0.001
HDL-C (mmol/L)	1.25 ± 0.22	1.02 (0.86–1.17)	0.24 (0.20–0.31)	< 0.001	0.493	< 0.001	< 0.001
LDL-C (mmol/L)	2.40 ± 0.51	2.17 ± 0.81	0.79 (0.38–1.26)	< 0.001	1.000	< 0.001	< 0.001
VLDL-C (mmol/L)	0.71 ± 0.20	0.82 (0.56–1.18)	0.73 (0.46–1.16)	0.345	NA	NA	NA
Glu (mmol/L)	4.56 ± 0.48	4.77 (4.36–5.34)	4.10 (3.37–5.27)	0.002	1.000	0.139	0.004
K^+^ (mmol/L)	4.12 ± 0.25	4.14 ± 0.31	4.05 ± 0.51	0.667	NA	NA	NA
Na^+^ (mmol/L)	143 ± 2	142 ± 2	139 (136–141)	< 0.001	0.436	< 0.001	< 0.001
Ca^2+^ (mmol/L)	2.27 (2.23–2.30)	2.25 ± 0.11	2.08 (2.01–2.16)	< 0.001	1.000	< 0.001	< 0.001
WBC (10^9^/L)	6.07 (5.45–6.67)	5.13 ± 1.64	7.9 (5.55–10.57)	< 0.001	0.184	0.007	< 0.001
Neutrophil (10^9^/L)	3.42 (2.96–3.95)	2.51 (1.69–3.50)	5.55 (3.57–8.1)	< 0.001	0.208	< 0.001	< 0.001
RBC (10^9^/L)	4.57 ± 0.55	4.69 ± 0.56	3.95 ± 0.65	< 0.001	1.000	< 0.001	< 0.001
HB (g/L)	138 (128–153)	146 ± 15	123 ± 18	< 0.001	0.753	< 0.001	< 0.001
PLT(10^9^/L)	232 (202–265)	187 ± 62	91 (67–127)	< 0.001	0.188	< 0.001	< 0.001

Data are presented as means ± standard deviations (SD) for normally distributed variables, medians with interquartile ranges (IQR) for non-normally distributed variables, and frequencies (n) with percentages (%) for categorical variables

*ALB* albumin, *ALT* alanine aminotransferase, *AST* aspartate aminotransferase, *ALP* alkaline phosphatase, *ChE* cholinesterase, *TBA* total bile acids, *TBil* total bilirubin, *DBil* direct bilirubin, *IBil* indirect bilirubin, *ADA* adenosine deaminase, *GGT* gamma-glutamyl transferase, *GFR* glomerular filtration rate, *Cr* creatinine, *BUN* blood urea nitrogen, *TG* triglycerides, *TC* total cholesterol, *HDL-C* high-density lipoprotein cholesterol, *LDL-C* low-density lipoprotein cholesterol, *VLDL-C* very low-density lipoprotein cholesterol, *Glu* blood glucose, *WBC* white blood cell count, *RBC* red blood cell count, *HB* hemoglobin, *PLT* platelet count, *NA* not applicable

Pairwise post-hoc comparisons were not performed if the corresponding omnibus test showed no significant difference. Adjusted *p*-values greater than 1.000 were truncated to 1.000 for presentation

*p*^a^: *p* value of overall comparisons among HC, CHB and ACLF patients

*p*^b^,* p*^c^,* p*^d^: pairwise post-hoc comparisons of CHB vs. HC, ACLF vs. HC, CHB vs. ACLF, respectively

Serum levels of ALT, AST, ALP, ADA, GGT, and TG were significantly elevated in CHB patients compared to HCs, reflecting active liver inflammation and metabolic changes. In comparison to CHB patients, HBV-ACLF patients showed significantly higher levels of ALP, TBA, TBil, DBil, IBil, ADA, WBC, and neutrophils. Conversely, HBV-ACLF patients demonstrated markedly reduced levels of ALB, ChE, GGT, Cr, TC, HDL-C, LDL-C, Glu, Na⁺, Ca^2^⁺, RBC, HB, and PLT counts.

Additionally, the 125 enrolled HBV-ACLF patients were categorized into ACLF-1, ACLF-2, and ACLF-3 according to COSSH criteria (49 at ACLF-1, 47 at ACLF-2, and 29 at ACLF-3), with detailed clinical characteristics provided in Supplementary Table S2. There were no significant differences in age and gender distribution, HBV-DNA levels, HBeAg positivity rate, or cirrhosis prevalence across the different HBV-ACLF stages. Compared to ACLF-1, patients in ACLF-2 exhibited significant increases in IBil, INR, and PT, along with significant decreases in TBA, TG, and VLDL-C. Advancing to ACLF-3, these trends persisted, with additional elevations noted in ChE, LDL-C, D-dimer, WBC, neutrophil, and ferritin levels.

### Metabolic analysis of HBV-ACLF

#### Metabolic profiling of serum of CHB and HBV-ACLF patients

This work aimed to elucidate the dynamic metabolic alterations among different stages of HBV-ACLF, diagnosed based on the COSSH-ACLF guidelines. We utilized CIL LC–MS to profile the amine/phenol submetabolomes in serum samples from CHB, HBV-ACLF patients, as well as healthy controls (HCs). A total of 2053 peak pairs were detected (Supplementary Table S1). The identification of detected peak pairs were categorized into three tiers of identification: tier 1 (positive identification), tier 2 (high-confidence putative identification), and tier 3 (putative identification). Specifically, there were 208, 255, and 1267 peak pairs identified as tier 1, tier 2, and tier 3, respectively. Thus, 1730 out of 2053 (84.3%) peak pairs were either identified or mass-matched to metabolite structures. The extensive coverage of CIL LC–MS enabled sensitive monitoring of metabolic dysregulations and facilitated the screening of biomarkers associated with short-term adverse outcomes in HBV-ACLF.

**Fig. 1:**
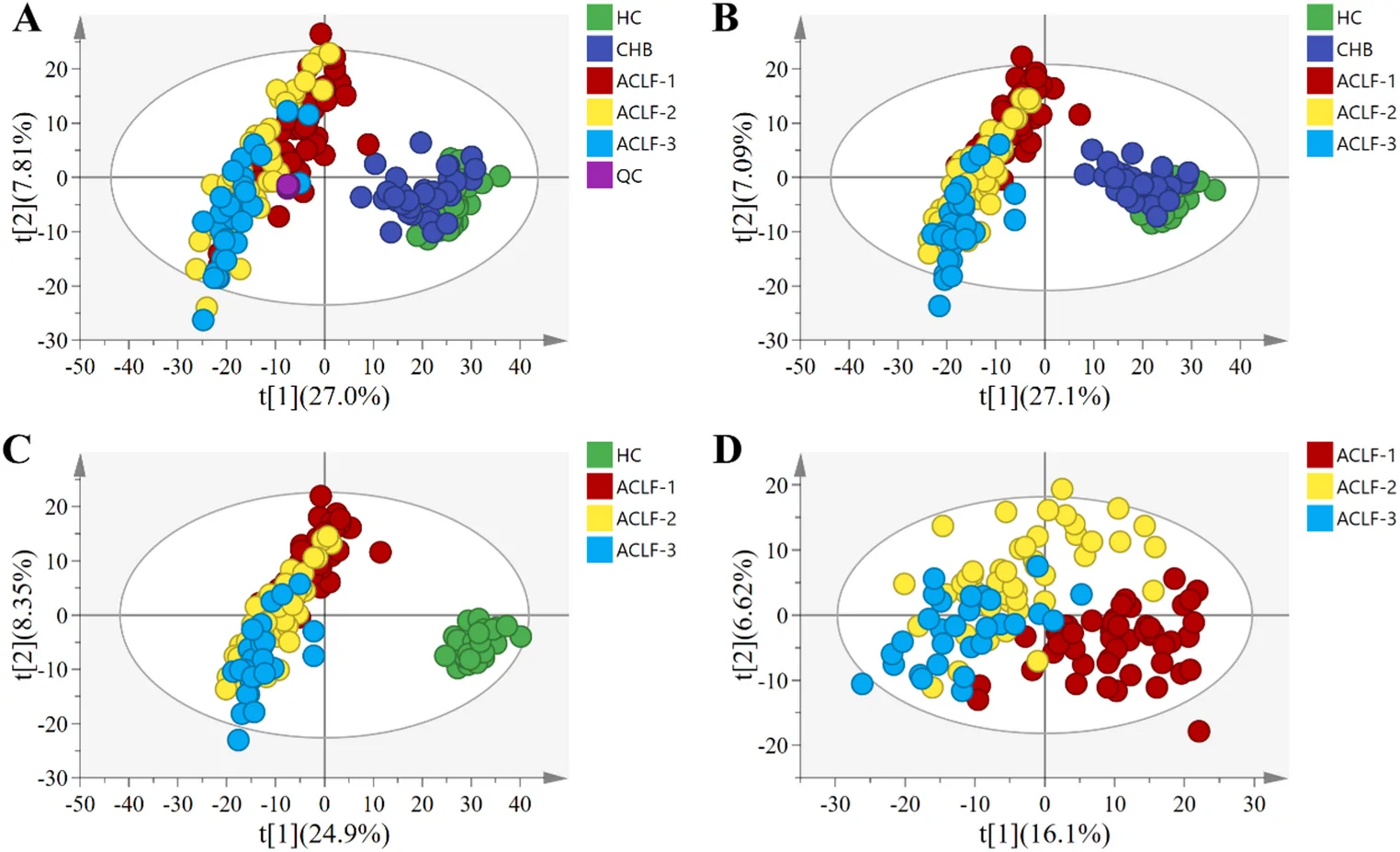
Multivariate analysis illustrating metabolic differences during the progression of HBV-ACLF. **A** PCA score plots, including quality control (QC) samples, with R^2^X = 0.697 and Q^2^ = 0.514. **B** PLS-DA score plots with R^2^X = 0.561 and Q^2^ = 0.721. **C** PLS-DA score plots comparing different stages of HBV-ACLF to healthy controls (HCs). **D** PLS-DA score plots for different stages of HBV-ACLF

To investigate the metabolic differences during the progression of HBV-ACLF, we performed multivariate analyses based on PCA and PLS-DA. The PCA score plots illustrated that HBV-ACLF groups (ACLF-1, ACLF-2, and ACLF-3) clustered together, distinctly separated from the CHB and HCs. The tight clustering of the quality control (QC) samples indicated excellent technical stability (Fig. 1A). After excluding QC samples, PLS-DA score plots confirmed that the metabolic differences between HBV-ACLF and HCs were considerably more pronounced than those observed between CHB and HCs (Fig. 1B). While some overlap exists, general metabolic shifts could be discerned across different stages of HBV-ACLF when CHB and HCs were sequentially excluded (Fig. 1C, D).

#### Significant metabolites associated with HBV-ACLF

**Fig. 2:**
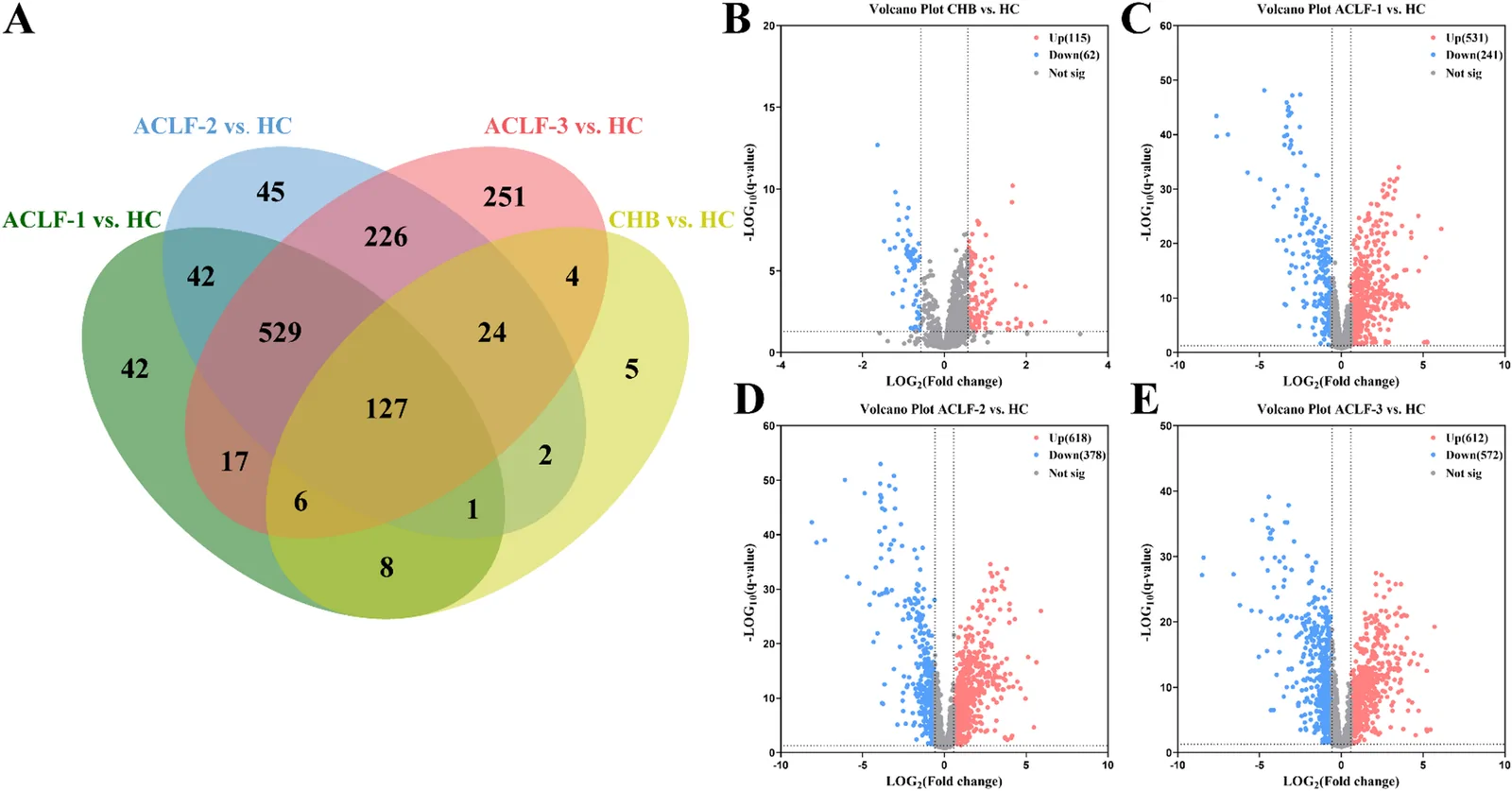
Univariate analysis of significant metabolites from binary comparisons between HBV-ACLF and CHB groups against HCs. **A** Venn diagram displaying the count of significant metabolites across groups. **B**–**E** Volcano plots comparing CHB vs. HCs, ACLF-1 vs. HCs, ACLF-2 vs. HCs, and ACLF-3 vs. HCs, respectively. Significant metabolites were defined by a q-value < 0.05 and a fold change of either ≥ 1.5 or ≤ 0.67

To identify the metabolites associated with the progression of HBV-ACLF, we conducted binary comparisons of the CHB, ACLF-1, ACLF-2, and ACLF-3 groups against the HCs, respectively. Significant metabolites were screened out using a fold change threshold (≥ 1.5 or ≤ 0.67), alongside a q-value threshold of < 0.05. As illustrated by the Venn diagram (Fig. 2A) and volcano plots (Fig. 2B–E), there were 177, 772, 996, and 1184 significant metabolites in CHB, ACLF-1, ACLF-2, and ACLF-3 groups, respectively. This trend clearly demonstrates that the number of differential metabolites increases with the severity of the disease, indicating a progressive metabolic disturbance as patients’ transition from chronic hepatitis to more advanced stages of liver failure.

To address the gap in understanding metabolic changes, we further investigated the differences in serum metabolites using ^12^C-/^13^C-dansylation labeling LC–MS. Our study particularly focused on the amine and phenol submetabome, which are crucial for many metabolic pathways and account for over 49% and 43% of the metabolites documented in the Human Metabolome Database (HMDB) and the Kyoto Encyclopedia of Genes and Genomes (KEGG), respectively (Zhao et al., 2019). Notably, the CIL LC–MS strategy demonstrated high sensitivity and broad metabolic coverage, with 84.3% of the detected peak pairs being either positively identified or mass-matched.

In our further analysis of these differential metabolites, we focused specifically on tier 1 and tier 2 metabolites. These classifications reflect positive or high-confidence putative identifications, enhancing the reliability of our findings. By concentrating on these metabolites, we aim to identify those that may serve as robust biomarkers for disease progression, thereby unraveling the underlying mechanisms that drive metabolic alterations in HBV-ACLF. This layered understanding of the metabolome will facilitate the development of targeted therapeutic strategies and improve patient management in the context of liver disease.

**Fig. 3:**
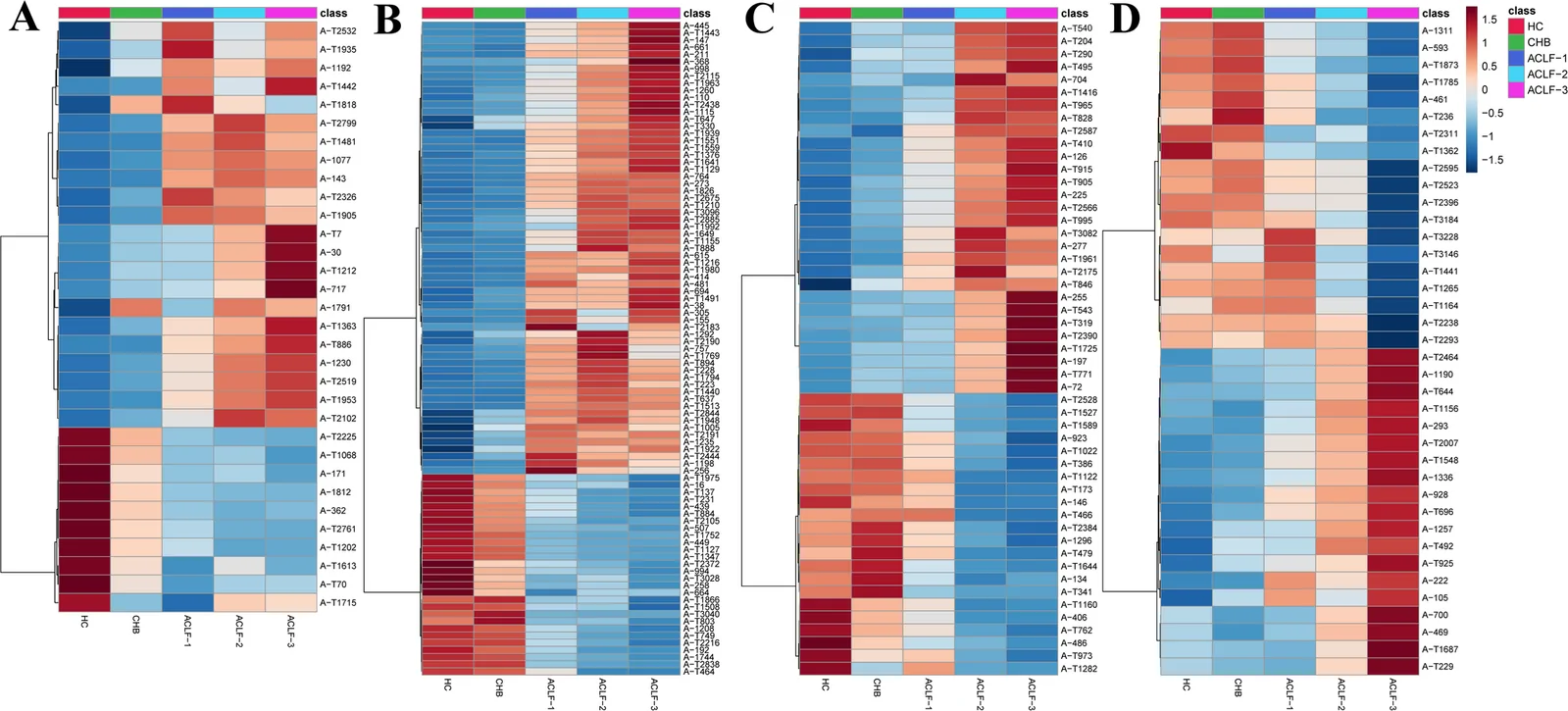
Heatmaps of significant metabolites with positive or high-confidence putative identification. **A** Metabolite subset 1: metabolites significantly altered across CHB, ACLF-1, ACLF-2, and ACLF-3. **B** Metabolite subset 2: metabolites that did not significantly change in CHB but were significantly altered in ACLF-1, ACLF-2, and ACLF-3. **C** Metabolite subset 3: metabolites significantly altered only in ACLF-2 and ACLF-3. **D** Metabolite subset 4: metabolites significantly altered only in ACLF-3

We categorized the identified significant metabolites into four subsets to illustrate the metabolic shifts that occur as the disease progresses. Metabolite subset 1 included significant metabolites present in all groups (CHB, ACLF-1, ACLF-2, and ACLF-3), reflecting shared metabolic perturbations. Subset 2 comprised metabolites that did not show significant changes in CHB but were significantly altered in ACLF-1, ACLF-2, and ACLF-3, indicating metabolic changes emerging from the ACLF-1 stage. Metabolite subset 3 included significant metabolites that arose specifically from the ACLF-2 stage, highlighting distinct metabolic alterations at this level of disease severity. Lastly, subset 4 contained metabolites unique to the ACLF-3 stage, recognizing the advanced state of liver dysfunction. Detailed information of each subset is provided in Supplementary Table S3, which includes 32, 91, 51, and 38 metabolites in subsets 1 to 4, respectively. Heatmaps presented in Fig. 3 illustrate the trends of these significant metabolites throughout disease progression.

Notably, dipeptides constituted a significant proportion of the differentially expressed metabolites in our study. A summary of the numbers and cumulative totals of dipeptides that significantly increased or decreased during the progression of HBV-ACLF is shown in Fig. S1, further underscoring their relevance in the metabolic landscape of the disease. The number of proteinogenic dipeptides that significantly increased was greater than those that decreased, confirming ongoing proteolysis from the perspective of dipeptides (Fig. S1A). Besides, we found that eight proteinogenic dipeptides were significantly raised, while five were significantly lower in CHB. These changes continued throughout the progression of HBV-ACLF, suggesting that abnormal protein catabolism may have started even before the onset of HBV-ACLF (Supplementary Table S3). Since dipeptides serve not only as intermediates in protein breakdown but also possess bioactive properties, such as antioxidant and immune-regulating activities, the changes in dipeptide metabolism deserve further investigation to determine their role in the immune response during HBV-ACLF.

Additionally, the increase in γ-glutamyl dipeptides, which are byproducts of glutathione metabolism, further correlates with the observed metabolic disturbances (Fig. S1B). These dipeptides can be formed through the activity of γ-glutamyl-cysteine synthetase and γ-glutamyltransferase (Ikeda & Fujii, 2023), indicating of enhanced glutathione synthesis and increased consumption during oxidative stress associated with liver dysfunction. The elevation of γ-glutamyl dipeptides has also been observed in patients with asymptomatic HBV infection and CHB (Soga et al., 2011). This trend may suggest that the body is attempting to cope with increased oxidative stress and inflammatory responses, both of which are critical in the progression of HBV-ACLF.

#### Metabolic pathways related to the severity of HBV-ACLF

**Fig. 4:**
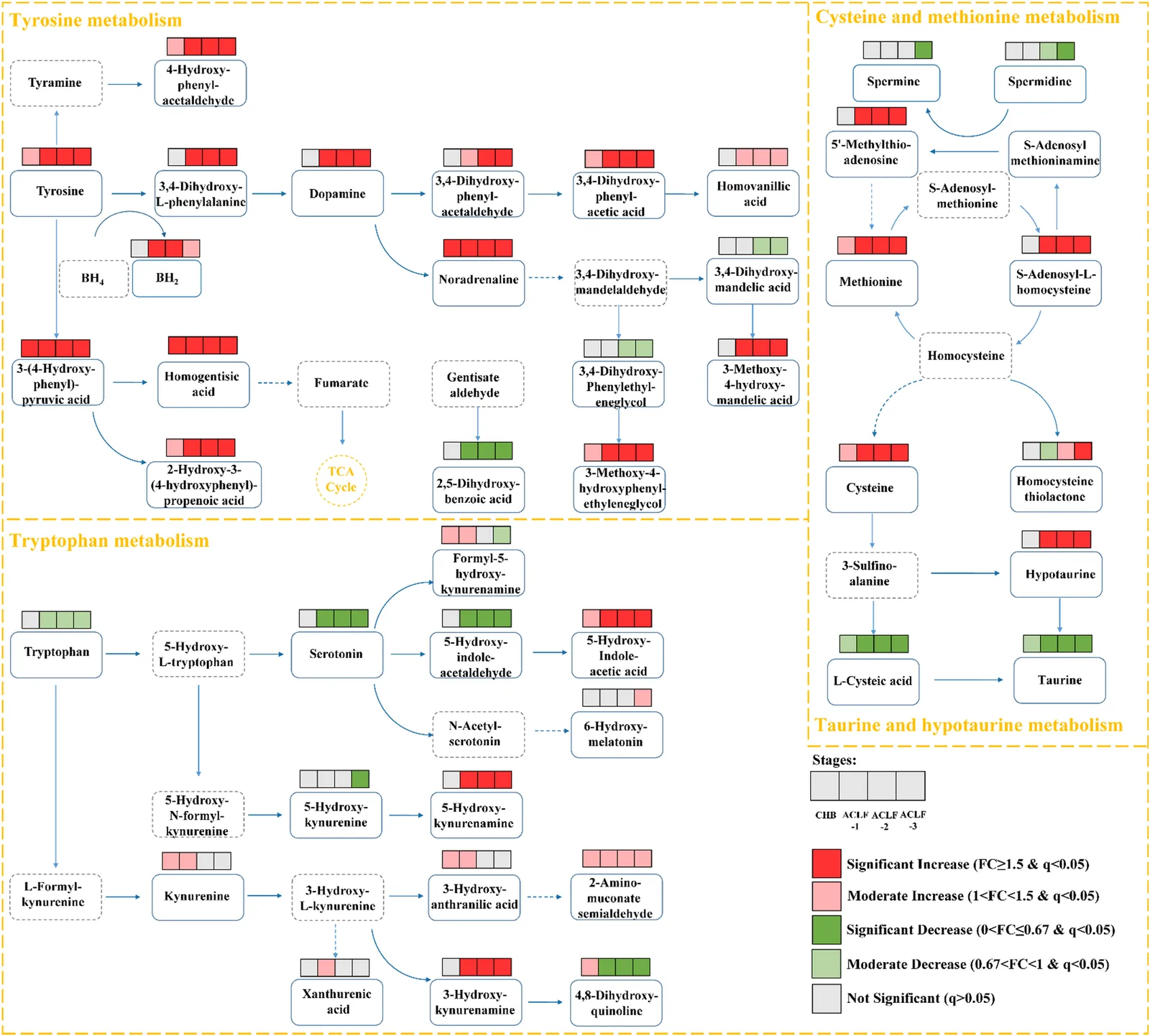
Schematic illustration of the most significantly altered metabolic pathways. Small squares above each rounded rectangle indicate the stages of HBV-ACLF at which the corresponding metabolites were altered. The fold change and significance of alterations are denoted by color: red and light red represent increases, while green and light green signify decreases. Metabolites enclosed in dashed rounded rectangles were not detected by CIL LC–MS. Solid arrows indicate single-step reactions, whereas dashed arrows represent multi-step reactions

Biological interpretation of the metabolic alterations in HBV-ACLF was achieved through pathway analysis of the metabolites from subsets 1–4. Based on the criteria of *p* < 0.05 and impact > 0, there were 14 metabolic pathways significantly affected (Fig. S2, Supplementary Table S4). Tyrosine metabolism, taurine and hypotaurine metabolism, tryptophan metabolism and cysteine and methionine metabolism were metabolic pathways most related to the development of HBV-ACLF. As schematically illustrated in Fig. 4, the usage of CIL-MS enabled us to sensitively monitor the dynamic changes in the aforementioned four metabolic pathways throughout the course of HBV-ACLF.

## Discussion

### Clinical insights into HBV-ACLF progression

In our cohort, laboratory indicators revealed that compared to CHB patients, those with HBV-ACLF exhibited significant accumulation of bile acids, bilirubin, ALP, and ADA, along with significant reductions in ALB, ChE, and GGT. This panel of biomarkers not only illustrates the extent of liver dysfunction but also serves as an early warning system for clinicians to identify patients at risk for acute deterioration. Elevated bile acids and bilirubin signify disrupted hepatic function, indicating cholestasis and jaundice, respectively, which are critical signs of liver failure (Farooqui et al., 2022).

The observed increase in WBC and neutrophils indicates an enhanced systemic inflammatory response, a hallmark of HBV-ACLF (Wu et al., 2018b). This systemic inflammation may not only exacerbate liver injury but also create a feedback loop that leads to further hepatic damage, complicating patient management. The link between inflammation and liver injury emphasizes the necessity for interventions aimed at mitigating these systemic effects. Additionally, the decrease in TC, HDL-C, LDL-C, and blood glucose suggests significant metabolic derangement. These findings indicate a compromised ability of the liver to regulate energy and lipid metabolism, potentially leading to malnutrition and cachexia, both of which are prevalent among patients with liver failure (Cai et al., 2024; Zhang et al., 2023).

As the disease progresses from ACLF-1 to ACLF-3, coagulation function deteriorates, accompanied by an exacerbation of inflammatory responses. Detailed analysis of laboratory indicators in patients at different stages shows that although these indicators exhibit changes, most of the laboratory indicators of viral infection, hepatic and renal function, and electrolytes were incapable in differentiating stages of HBV-ACLF. This indicates that traditional laboratory indicators are not effective in accurately determine the specific clinical stage of patients, highlighting the need for new biomarkers to assist with early identification and more personalized treatment.

Additionally, our study identified a significant gender disparity in the prevalence of HBV-ACLF, with a higher proportion of male patients affected. This difference may be attributed to biological factors, including hormonal variations, genetic predispositions, and immune response differences that increase males’ vulnerability to severe liver disease. Brown et al. discuss how sexual dimorphism in chronic HBV infection can affect disease outcomes, noting that males typically experience more severe disease progression compared to females (Brown et al., 2022). This is due to several factors, such as higher viral load and the impact of hormonal differences on immune responses, which can influence susceptibility to liver damage. Understanding these factors is crucial for addressing this disparity and for developing appropriate targeted interventions. By tailoring strategies that consider these biological differences, healthcare providers can improve outcomes for male patients with HBV-ACLF and enhance overall management of the disease.

### Metabolic changes and pathway alterations associated with HBV-ACLF

With stringent quality control, we quantified 2053 metabolites in healthy controls, CHB, and COSSH‑defined ACLF‑1/‑2/‑3 to systematically map stage-resolved metabolic alterations. The findings provide significant insights into the complex interplay of metabolic dynamics throughout disease progression and its pathophysiological consequences. The marked differences in serum metabolites between CHB and various stages of HBV-ACLF highlight the metabolic disturbances that arise as liver function deteriorates. Beyond their discriminatory value, these metabolic alterations may also provide mechanistic clues linking hepatic insufficiency to systemic inflammation, oxidative stress, and immune dysregulation in HBV-ACLF.

One of the most notable observations was the increase in circulating amino acids and dipeptides, reflecting disrupted protein homeostasis in the setting of hepatic injury. More specifically, these changes likely reflect the combined effects of impaired hepatic amino acid utilization, reduced metabolic clearance, and a hypercatabolic state associated with systemic inflammation. In this context, the accumulation of circulating amino acids and dipeptides may serve as a metabolic readout of both impaired hepatic metabolic capacity and enhanced peripheral proteolysis. The predominance of increased, rather than decreased, proteinogenic dipeptides further supports the notion that abnormal protein catabolism is a hallmark of HBV-ACLF progression and suggests that these alterations may begin before overt ACLF develops. This observation also underscores the potential of dipeptides as early biomarkers of disease progression.

The significant changes in dipeptide metabolism may have biological relevance beyond protein breakdown alone. In addition to serving as intermediates of proteolysis, certain dipeptides have been reported to exert antioxidant and immune-regulatory effects (Martin et al., 2020; Saadati et al., 2024). Accordingly, altered dipeptide profiles in HBV-ACLF may not merely reflect passive catabolism, but may also participate in shaping the inflammatory milieu. This possibility warrants further investigation in future mechanistic studies.

The observed increase in γ-glutamyl dipeptides provides additional insight into the oxidative stress responses associated with HBV-ACLF. Because γ-glutamyl transfer reactions are closely linked to glutathione turnover, the elevation of these metabolites may indicate accelerated glutathione consumption and recycling under conditions of persistent oxidative injury. This interpretation is further supported by the concurrent perturbation of sulfur-containing amino acid metabolism, suggesting that redox imbalance is an integral component of HBV-ACLF progression rather than a secondary bystander effect. Collectively, these findings point to increased reliance on glutathione-related antioxidant defenses during progressive liver injury.

At the pathway level, our results indicate coordinated remodeling of tyrosine, tryptophan, cysteine/methionine, and taurine/hypotaurine metabolism during HBV-ACLF progression. Among these, one of the most prominent changes was the substantial accumulation of tyrosine and its metabolic byproducts in serum. We identified 17 metabolites associated with tyrosine metabolism, of which 13 were significantly elevated. This marked accumulation of tyrosine likely arises from intensified proteolysis and impaired mitochondrial amino acid-ketone body conversion pathways (Zaccherini et al., 2021), reflecting the liver’s inability to maintain normal metabolic functions. Given the central role of the liver in aromatic amino acid handling, these findings are biologically consistent with impaired hepatic catabolic capacity during ACLF progression.

Tyrosine hydroxylase (TH) catalyzes the conversion of tyrosine to 3,4-dihydroxy-L-phenylalanine (L-DOPA), while simultaneously oxidizing its coenzyme tetrahydrobiopterin (BH_4_) to 7,8-dihydrobiopterin (BH_2_) (Wang et al., 2021). The significant increase in BH_2_ levels observed in ACLF-1 and ACLF-2 corresponds with elevated dopamine, L-noradrenaline, and its terminal metabolite 3-methoxy-4-hydroxyphenylethyleneglycol, indicating enhanced catecholamine synthesis through the tyrosine-DOPA pathway. These changes may reflect activation of systemic stress-response pathways during severe inflammation and organ dysfunction (Weiss et al., 2023), thereby providing a plausible link between liver failure, neuroendocrine adaptation, and systemic metabolic reprogramming. Additionally, the accumulation of homogentisic acid, a precursor of fumarate in the tricarboxylic acid (TCA) cycle, may further indicate impaired entry of tyrosine-derived intermediates into central energy metabolism. This finding further supports the concept that HBV-ACLF is associated with impaired substrate utilization and disordered energy homeostasis.

In addition to tyrosine metabolism, we observed significant perturbations in tryptophan metabolism. Tryptophan 2,3-dioxygenase (TDO), produced in the liver, accounts for approximately 90% of tryptophan catabolism (Klaessens et al., 2022). Our analysis of 14 tryptophan-related metabolites revealed distinct variation patterns across different branches of tryptophan catabolism throughout the progression of HBV-ACLF. Notably, metabolites in the tryptophan-kynurenine pathway, such as kynurenine, 3-hydroxy-anthranilic acid, and 2-amino-muconate semialdehyde, demonstrated moderate upregulation as early as the CHB stage, with pronounced elevation of 3-hydroxykynurenamine (3-HKA) beginning at ACLF-1. This pattern suggests that inflammatory tryptophan degradation is activated before full-blown ACLF and becomes more prominent with disease progression.

Emerging evidence suggests that 3-HKA acts as an immunosuppressive regulator through IFN-γ-mediated STAT1/NF-κB pathways (Clement et al., 2021), underscoring its potential as a biomarker for disease progression. Prior studies have quantified levels of kynurenine, kynurenic acid, and quinolinic acid, demonstrating activation of the tryptophan-kynurenine pathway in patients with acute decompensation, with or without ACLF, further supporting our findings (Claria et al., 2019). Mechanistically, activation of the kynurenine pathway may have several consequences. On the one hand, it may support NAD^+^ biosynthesis to meet increased metabolic demands under inflammatory stress. On the other hand, several kynurenine-derived metabolites are linked to immunomodulation, oxidative injury, and neurotoxicity. Therefore, the observed activation of this pathway may reflect not only a metabolic adaptation to liver failure but also a potential contributor to immune dysfunction and systemic complications in HBV-ACLF.

The tryptophan-serotonin branch also showed evidence of remodeling, with enhanced serotonin turnover as reflected by increased 5-hydroxyindoleacetic acid (5-HIAA) beginning at ACLF-1, alongside reductions in intermediates such as serotonin and 5-hydroxyindoleacetaldehyde. This pattern suggests a shift in serotonin dynamics that may have important implications for immune regulation and vascular function in HBV-ACLF. Peripheral serotonin plays a vital role in regulating immune cells, hepatic energy metabolism, and platelet activation (Banskota & Khan, 2022; Moon et al., 2022; Rieder et al., 2021). In addition, 5-HIAA acts as an endogenous ligand for the G protein-coupled receptor GPR35, facilitating neutrophil recruitment to sites of inflammation and linking metabolic alterations with immune response in ACLF, particularly in advanced stages (De Giovanni et al., 2022)**.** Taken together, the reciprocal pattern of kynurenine-pathway activation and serotonin-pathway suppression suggests a broader rerouting of tryptophan metabolism toward inflammatory and immunoregulatory processes during HBV-ACLF progression.

Furthermore, our findings indicate activation of cysteine and methionine metabolism in HBV-ACLF, as evidenced by significant increases in methionine, S-adenosyl-L-homocysteine (SAH), 5ʹ-methylthioadenosine (5ʹ-MTA), and cysteine beginning at ACLF-1. Because these metabolites are closely linked to methyl-group transfer, transsulfuration, and glutathione-related redox regulation, their elevation may reflect an adaptive response to oxidative stress and increased biosynthetic demand during systemic inflammation. The substantial increase in adenosine (Supplementary Table S3, metabolites subset 1) is indicative of this process, emphasizing the interconnected roles of these amino acids in energy metabolism and cellular signaling pathways in liver dysfunction.

Additionally, taurine acts as an indirect antioxidant by improving the function of the electron transport chain to prevent accumulation of superoxide (Jong et al., 2012). The activation of taurine and hypotaurine metabolism may serve to compensate for the depletion of taurine as oxidative stress increases during the progression of HBV-ACLF, emphasizing the importance of antioxidant mechanisms in liver health. Together with the γ-glutamyl and methionine-cycle changes described above, these data support the notion that antioxidant defense pathways are actively remodeled during HBV-ACLF, likely in an attempt to preserve redox homeostasis despite progressive hepatic injury.

Overall, the pathway-level metabolic alterations observed in this study are consistent with a model in which impaired hepatic metabolic capacity, enhanced proteolysis, oxidative stress, and immune dysregulation evolve in parallel during HBV-ACLF progression. These changes are unlikely to represent isolated biochemical events; rather, they appear to be interconnected components of the systemic response to severe liver failure. Nevertheless, because our study is based on retrospective serum metabolomics, these mechanistic interpretations should be considered biologically informed associations rather than direct evidence of causality. Future experimental and translational studies will be needed to determine how these metabolic pathways functionally contribute to liver injury, immune dysfunction, and clinical deterioration in HBV-ACLF.

## Conclusion

In conclusion, this study provides a high-coverage characterization of serum metabolic alterations across the progression from healthy controls to CHB and then to different stages of HBV-ACLF using chemical isotope labeling LC–MS. We identified distinct stage-related metabolic trajectories, with progressive disturbances in amino acid and dipeptide metabolism accompanying disease deterioration. Among the most prominent changes were the accumulation of tyrosine-related metabolites, activation of the tryptophan-kynurenine pathway, suppression of the tryptophan-serotonin branch, and remodeling of cysteine, methionine, taurine, and γ-glutamyl-related metabolism.

These findings support a model in which impaired hepatic metabolic capacity, enhanced proteolysis, oxidative stress, and immune dysregulation evolve in parallel during HBV-ACLF progression. While conventional laboratory indicators can distinguish CHB from HBV-ACLF, they remain insufficient for accurately stratifying ACLF stages, underscoring the potential value of metabolomic markers for disease monitoring and patient stratification.

Overall, our study strengthens the biological relevance of metabolic dysregulation in HBV-ACLF and provides a framework for understanding how liver failure is linked to systemic inflammatory and redox-related responses. These metabolite signatures and pathway alterations may facilitate the development of novel biomarkers and therapeutic targets. Nevertheless, because the present study is based on retrospective serum metabolomic profiling, further experimental and prospective clinical studies are needed to validate the mechanistic and clinical significance of these findings.

## Supplementary Information

Below is the link to the electronic supplementary material.

Supplementary Material 1

Supplementary Material 2

Supplementary Material 3

Supplementary Material 4

## Data Availability

According to reasonable requirements, data can be obtained from the first author.
