# Supplementary material for: Characterization of multi-stage metabolic alterations in hepatitis B virus-related acute-on-chronic liver failure using high-coverage metabolomics
Source: Metabolomics. 2026 Jun 16;22(4):93. doi: 10.1007/s11306-026-02462-0 (PMC13272236; doi:10.1007/s11306-026-02462-0)
Supplement: Supplementary file 2 — Supplementary Material 2 [file 11306_2026_2462_MOESM2_ESM.docx]

**Characterization of Multi-Stage Metabolic Alterations in Hepatitis B Virus-Related Acute-on-Chronic Liver Failure Using High-Coverage Metabolomics**

**Zhehua Zhang^1,2 †^, He Jiang^1,2†^, Guoqi Zhang^3^, Deying Chen^1,2^, Xiaoling Su^1,2^, Liang Li^4^, Lanjuan Li^1,2*^**

^1^ State Key Laboratory for Diagnosis and Treatment of Infectious Diseases, National Clinical Research Center for Infectious Diseases, China-Singapore Belt and Road Joint Laboratory on Infection Research and Drug Development, National Medical Center for Infectious Diseases, Collaborative Innovation Center for Diagnosis and Treatment of Infectious Diseases, The First Affiliated Hospital, Zhejiang University School of Medicine, Hangzhou, China

^2^ Yuhang Institute of Medical Science Innovation and Transformation, Hangzhou, China

^3^ Department of Critical Care Medicine, Shulan (Hangzhou) Hospital Affiliated to Zhejiang Shuren University Shulan International Medical College, Hangzhou, China

^4^The Metabolomics Innovation Centre and Department of Chemistry, University of Alberta, Edmonton, AB, Canada

^†^ These authors contributed equally

^*^ Corresponding author: Lanjuan Li, E-mail: ljli@zju.edu.cn

**Contents:**

1. **Supplemental Note.S1.** Patients and clinical characteristics
2. **Supplemental Note.S2.** Experimental methods
3. **Supplemental Figure. S1.** Variation patterns of significant dipeptides
4. **Supplemental Figure. S2.** Pathway analysis of significant metabolites screened from different stages of HBV-ACLF.
5. **Supplemental Table S1.** List of peak pairs detected from CIL LC-MS measurement of the samples (In Excel).
6. **Supplemental Table S2.** Clinical characteristics of ACLF-1, ACLF-2 and ACLF-3 patients according to the guidelines of COSSH-ACLF
7. **Supplemental Table S3.** Significant metabolites occurred in different stages of HBV-ACLF (In Excel).
8. **Supplemental Table S4.** Summary of pathway analysis results for HBV-ACLF.

**Supplemental Note.S1. Patients and clinical characteristics**

**Inclusion and exclusion criteria:**

The inclusion criteria of HCs were as follows: no history of viral hepatitis, exhibited no abnormal symptoms, no history of organic diseases (e.g., heart, liver, kidneys) and no chronic illness (e.g., hypertension, diabetes mellitus).

The inclusion criteria of CHB patients were as follows: a history of hepatitis B or plasma HBsAg positivity ≥6 months, alanine aminotransferase (ALT) or aspartate aminotransferase (AST) levels > 2x normal upper limit, evidence of inflammation based on liver biopsy.

The inclusion criteria of HBV-ACLF were based on the COSSH-ACLF criteria (Wu *et al.*, 2018): patients with HBV etiology who present TBil ≥ 12 mg/dL and an INR ≥ 1.5, regardless of the presence of cirrhosis. Based on this criteria, patients were further classified into ACLF-1, ACLF-2, and ACLF-3. ACLF-1 patients included: (1) kidney failure alone; (2) single liver failure accompanied by an INR ≥ 1.5, and/or kidney dysfunction, and/or hepatic encephalopathy (HE) Grade I–II; (3) a single organ failure involving the coagulation, circulatory, or respiratory system, combined with kidney dysfunction and/or HE Grade I–II; (4) cerebral failure alone with kidney dysfunction. ACLF-2 patients included failures of two organ systems. ACLF-3 patients included failures of three or more organ systems.

Exclusion criteria of HBV-ACLF included co-infection with other hepatitis viruses; concomitant of alcoholic liver disease, drug-induced hepatitis, autoimmune hepatitis, primary biliary cholangitis; history of hepatocellular carcinoma or cholangiocarcinoma; liver failure induced by HBV reactivation after liver transplantation.

**Clinical information collection：**

The general clinical characteristics of the study participants were retrieved from outpatient medical records. These data encompassed demographic information, including age and sex. Hepatic and renal function parameters were evaluated, covering albumin (ALB), alanine aminotransferase (ALT), aspartate aminotransferase (AST), alkaline phosphatase (ALP), total bile acids (TBA), total bilirubin (TBil), direct bilirubin (DBil), indirect bilirubin (IBil), gamma-glutamyl transferase (GGT), glomerular filtration rate (GFR), creatinine (Cr), blood urea nitrogen (BUN), cholinesterase (ChE), adenosine deaminase (ADA). HBV infection markers consisted of hepatitis B e-antigen (HBeAg) and HBV DNA levels. Hematological and coagulation profiles involved white blood cell count (WBC), neutrophil count, red blood cell count (RBC), hemoglobin (HB), and platelet count (PLT), international normalized ratio (INR), prothrombin time (PT), and D-dimer. Lipid and energy metabolism indicators included triglycerides (TG), total cholesterol (TC), high-density lipoprotein cholesterol (HDL-C), low-density lipoprotein cholesterol (LDL-C), very low-density lipoprotein cholesterol (VLDL-C), blood glucose (Glu); Electrolyte measurements comprised serum potassium (K^+^), sodium (Na^+^), and calcium (Ca^2+^). Additionally, biomarkers for hepatocyte regeneration and necrosis were assessed, such as alpha fetoprotein (AFP) and ferritin.

**Supplemental Note.S2. Experimental methods**

**Sample collection and preparation:**

Fasting venous blood (2 mL) was collected from eligible participants who met the inclusion and exclusion criteria using BD Vacutainer blood separator tubes (Becton, Dickinson and Company, USA). The tubes were allowed to stand at room temperature for 30 minutes, and centrifugated at 3500 rpm and 4°C for 10 minutes. The resulting supernatant serum was aliquoted and stored at - 80°C until analysis (Chen *et al.*, 2020).

All the serum samples were thawed on ice before analysis. To precipitate proteins, 90 μL prechilled methanol (Fisher Chemical, USA) was added into 30 μL serum, centrifuged at 15,000g for 30 minutes. The resulting supernatant was completely dried down using a SpeedVac vacuum concentrator (Labconco, USA). A 10 μL aliquot of serum from each individual sample was combined to create a pooled sample, which was processed identically following the same protein precipitation procedure.

**Dansylation labeling:**

Dansylation was carried out according to an established protocol (Wang *et al.*, 2024). Briefly, the dried extract of serum was dissolved in 25 μL of ultrapure water. The resulting solution was mixed with 12.5 μL of sodium carbonate/sodium bicarbonate buffer (0.5 M, pH 9.5) and 37.5 μL of acetonitrile containing either ^12^C-dansyl chloride or ^13^C-dansyl chloride. The derivatization reaction were performed at 40 ℃ for 45 min. Subsequently, 7.5 μL of NaOH solution (250 mM) was added, and incubated at 40 ℃ for another 10 min to remove excess dansyl chloride. Finally, formic acid (FA) solution in 1:1 ACN/H_2_O (30 μL) was added to acidify the solution.

**LC-MS parameters:**

LC-MS analysis was performed using Ultimate 3000 UHPLC (Thermo Scientific, USA) coupled with Impact II Q-TOF (Bruker, USA). A Waters BEH C18 column was selected (1.7 μm, 2.1 mm × 100 mm, Waters, USA). The mobile phases were water and acetonitrile, each containing 0.1% (v/v) formic acid (mobile phases A and B, respectively). The elution gradient started at 25% B, increased to 99% B at 10.00 min, held until 13.00 min, returned to 25% B at 13.10 min, and maintained the end of the run at 17 min. The separation was conducted at a flow rate of 0.4 mL/min with column temperature set at 50 °C. All the MS data were acquired in positive ion mode with a scan rate of 1 Hz (Zhang *et al.*, 2022). All samples were randomised for LC-MS analysis.

**References**

Chen, D., Han, W., Huan, T., Li, L. and Li, L. (2020) Effects of Freeze-Thaw Cycles of Blood Samples on High-Coverage Quantitative Metabolomics. *Anal Chem* **92,** 9265-9272. <https://doi.org/10.1021/acs.analchem.0c01610>

Wang, X., Shang, D., Chen, J., Cheng, S., Chen, D., Zhang, Z. *et al.* (2024) Serum metabolomics reveals the effectiveness of human placental mesenchymal stem cell therapy for Crohn's disease. *Talanta* **277,** 126442. <https://doi.org/10.1016/j.talanta.2024.126442>

Wu, T., Li, J., Shao, L., Xin, J., Jiang, L., Zhou, Q. *et al.* (2018) Development of diagnostic criteria and a prognostic score for hepatitis B virus-related acute-on-chronic liver failure. *Gut* **67,** 2181-2191. <https://doi.org/10.1136/gutjnl-2017-314641>

Zhang, Z., Chen, D., Yu, J., Su, X. and Li, L. (2022) Metabolic perturbations in human hepatocytes induced by bis (2-ethylhexyl)-2,3,4,5-tetrabromophthalate exposure: Insights from high-coverage quantitative metabolomics. *Anal Biochem* **657,** 114887. <https://doi.org/10.1016/j.ab.2022.114887>

**Figure S1.** Variation patterns of significant dipeptides (A) Changes of dipeptides throughout the progression of HBV-ACLF. The bars illustrated the number of significantly changed dipeptides at each stage of HBV-ACLF, whereas the scatter plots represented the cumulative changes of significantly changed proteinogenic dipeptides. (B) Box plot diagrams of significantly changed γ-glutamyl dipeptides. **p* < 0.05, ***p*< 0.01, ****p*<0.001 in comparison to HCs.


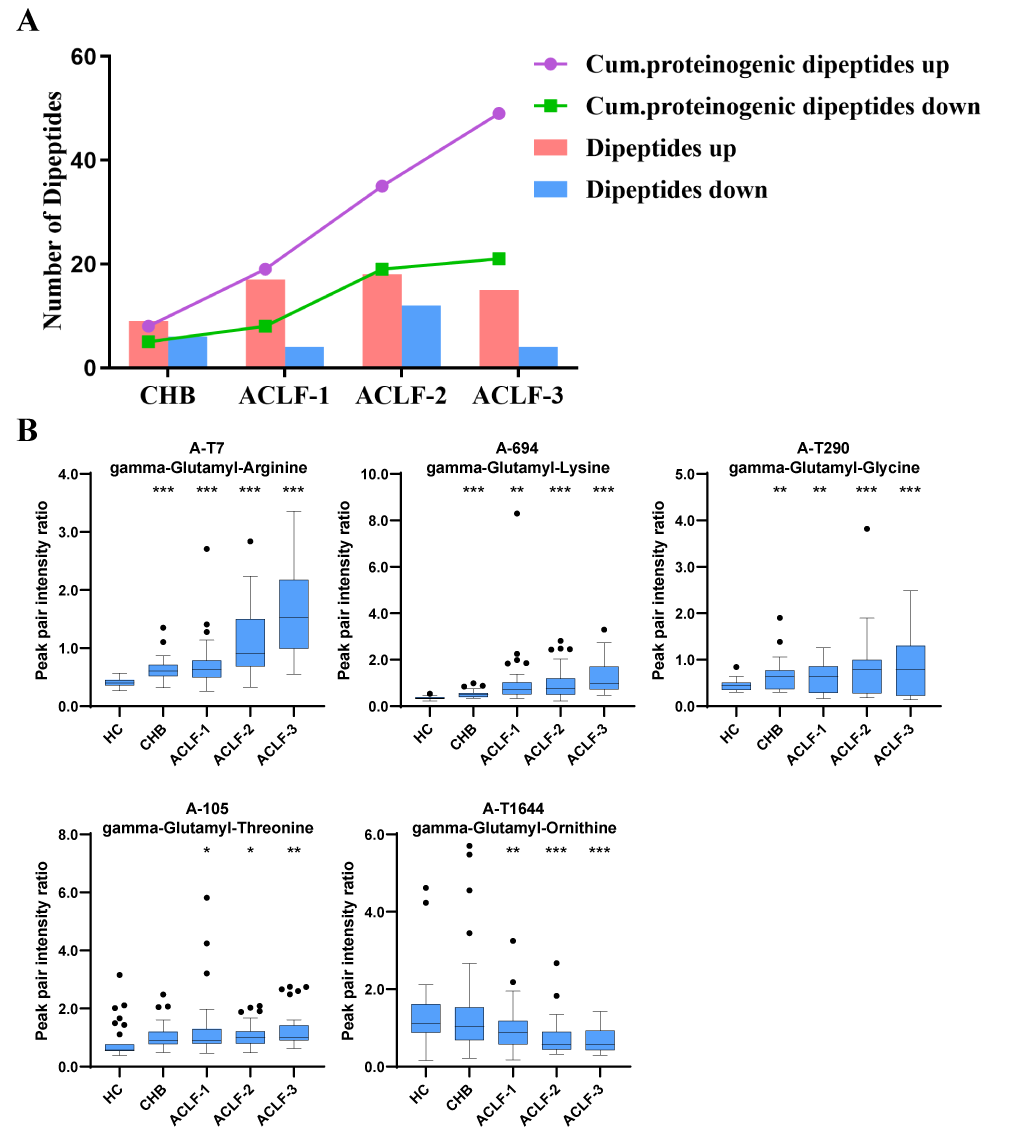


**Figure S2.** Pathway analysis of significant metabolites screened from different stages of HBV-ACLF.


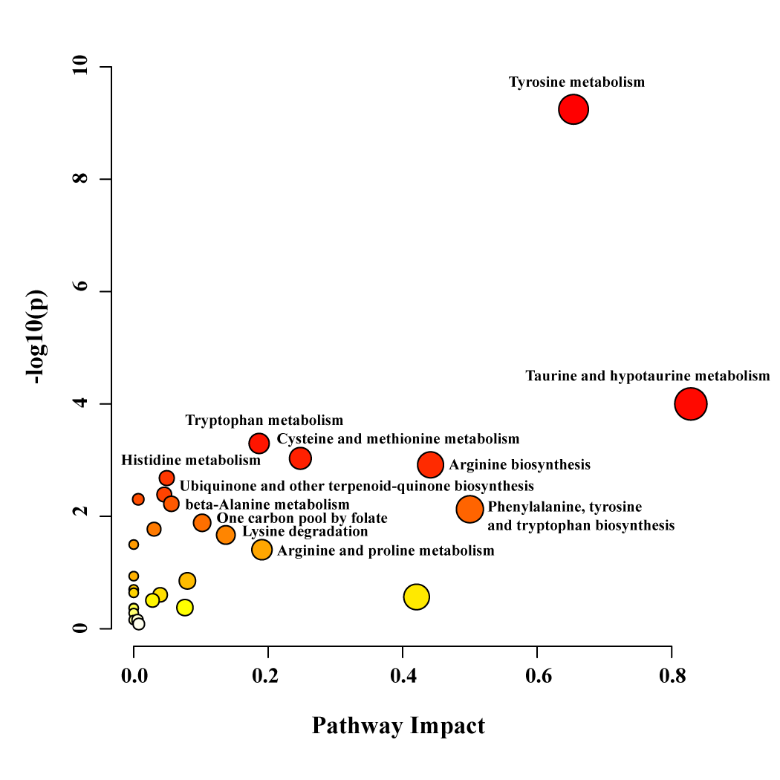


**Table S2.**Clinical characteristics of ACLF-1, ACLF-2 and ACLF-3 patients according to the guidelines of COSSH-ACLF

| Characteristics | ACLF-1(n=49) | ACLF-2(n=47) | ACLF-3(n=29) | *p^a^* value | *p^b^* value | *p^c^* value | *p^d^* value |
| --- | --- | --- | --- | --- | --- | --- | --- |
| Age (years) | 48±11 | 43(33-54) | 47(38-59) | 0.289 | NA | NA | NA |
| Male (%) | 43(87.8%) | 39(83.0%) | 25(86.2%) | 0.796 | NA | NA | NA |
| HBV-DNA (10^5^ IU/mL) | 3.7(1.2-754.0) | 15.6(8.5-2235.0) | 39.4(14.0-7920.0) | 0.108 | NA | NA | NA |
| HBeAg positive | 28(57.1%) | 22(46.8%) | 9(31.0%) | 0.083 | NA | NA | NA |
| Cirrhosis | 31(63.3%) | 35(74.5%) | 18(62.1%) | 0.403 | NA | NA | NA |
| ALB (g/L) | 31.2(28.8-32.7) | 30.4±2.8 | 31.1±3.5 | 0.727 | NA | NA | NA |
| ALT (U/L) | 233(103-400) | 294(131-504) | 330(177-628) | 0.219 | NA | NA | NA |
| AST (U/L) | 160(84-262) | 199(120-304) | 174(105-322) | 0.416 | NA | NA | NA |
| ALP (U/L) | 147±35 | 149±41 | 143±35 | 0.782 | NA | NA | NA |
| ChE (U/L) | 3566±1256 | 3705(2983-4662) | 5078±1860 | 0.001 | 0.862 | 0.001 | 0.024 |
| TBA (μmol/L) | 373.5(275.7-464.1) | 282.0(171.7-345.0) | 287.6±136.0 | 0.006 | 0.015 | 0.029 | 1.000 |
| TBil (μmol/L) | 346.6(276.5-450.7) | 351.8(311-472) | 391.6±81.3 | 0.306 | NA | NA | NA |
| DBil (μmol/L) | 277(223.6-384.8) | 249.8(218.8-348) | 252.1(208-355.5) | 0.282 | NA | NA | NA |
| IBil (μmol/L) | 61.1±22.5 | 100.0(79.0-123.0) | 118.7±42.2 | <0.001 | <0.001 | <0.001 | 1.000 |
| ADA (U/L) | 27(21.3-32) | 30.8(26.1-39) | 30.6(26.5-34.8) | 0.02 | 0.028 | 0.132 | 1.000 |
| GGT (U/L) | 88(60-105) | 70(46-106) | 57(42-86) | 0.078 | NA | NA | NA |
| GFR (mL/min) | 107.2±14.5 | 108.1±19.8 | 107.7(80.6-126.4) | 0.729 | NA | NA | NA |
| Cr (μmol/L) | 65±14 | 61(57-68) | 64(51-95) | 0.877 | NA | NA | NA |
| BUN (mmol/L) | 4.02(3.09-6.09) | 3.77(2.29-5.25) | 4.43(3.12-6.54) | 0.185 | NA | NA | NA |
| TG (mmol/L) | 1.18(0.98-1.60) | 0.96(0.83-1.20) | 1.01±0.22 | 0.001 | 0.002 | 0.017 | 1.000 |
| TC (mmol/L) | 2.02(1.63-2.40) | 1.75(1.35-2.19) | 2.22(1.60-3.16) | 0.019 | 0.097 | 1.000 | 0.028 |
| HDL-C (mmol/L) | 0.24±0.08 | 0.25(0.20-0.30) | 0.28(0.17-0.45) | 0.205 | NA | NA | NA |
| LDL-C (mmol/L) | 0.53(0.32-1.01) | 0.74(0.38-1.23) | 1.54±0.96 | <0.001 | 0.508 | <0.001 | 0.008 |
| VLDL-C (mmol/L) | 1.15±0.51 | 0.58(0.41-0.85) | 0.53±0.30 | <0.001 | <0.001 | <0.001 | 0.626 |
| Glu (mmol/L) | 3.95(3.45-4.83) | 3.89(3.05-4.98) | 5.00±1.89 | 0.162 | NA | NA | NA |
| K^+^ (mmol/L) | 4.11(3.69-4.31) | 4.01±0.56 | 4.23±0.51 | 0.165 | NA | NA | NA |
| Na^+^ (mmol/L) | 139±3 | 138±3 | 139±4 | 0.356 | NA | NA | NA |
| Ca^2+^ (mmol/L) | 2.10(2.04-2.18) | 2.05±0.11 | 2.07±0.14 | 0.132 | NA | NA | NA |
| INR | 1.87±0.23 | 2.96±0.45 | 3.12(2.75-3.61) | <0.001 | <0.001 | <0.001 | 0.607 |
| PT (s) | 20.5(19.3-23.5) | 33±5.3 | 35.2(30.6-39.6) | <0.001 | <0.001 | <0.001 | 0.579 |
| D-dimer (μg/L) | 2190(1249-2957) | 2341(1819-4936) | 4004(3007-5855) | <0.001 | 0.171 | <0.001 | 0.041 |
| WBC (10^9^/L) | 6.84(5.12-9.22) | 8.15±3.05 | 9.87±3.50 | 0.009 | 0.585 | 0.006 | 0.160 |
| Neutrophil (10^9^/L) | 4.36(3.27-6.99) | 5.83±2.69 | 7.54±3.04 | 0.008 | 1.000 | 0.006 | 0.076 |
| RBC (10^9^/L) | 3.92±0.61 | 3.96±0.70 | 4.00±0.65 | 0.876 | NA | NA | NA |
| HB (g/L) | 122±17 | 125±19 | 122±19 | 0.684 | NA | NA | NA |
| PLT (10^9^/L) | 94(64-122) | 86(69-127) | 103±44 | 0.935 | NA | NA | NA |
| AFP (ng/mL) | 103.56(40.79-288.2) | 55.35(23.15-152.20) | 43.95(15.93-141.73) | 0.072 | NA | NA | NA |
| Ferritin (ng/mL) | 2594.00(1150.90-3872.60) | 3618.40(1689.63-5337.60) | 4501.90(3002.25-7420.00) | 0.002 | 0.220 | 0.002 | 0.173 |

**Note:** The data are expressed as medians (IQR), mean ± (SD) or number of patients (%).*p*^a^: *p* value of overall comparisons among ACLF-1, ACLF-2 and ACLF-3 patients. *p*^b^, *p*^c^ , *p*^d^: pairwise post-hoc comparisons of ACLF-2 vs. ACLF-1, ACLF-3 vs. ACLF-1, ACLF-3 vs. ACLF-2, respectively. NA: Not applicable, post-hoc pairwise comparisons were not performed if if the corresponding omnibus test showed no significant difference. Adjusted *p*-values greater than 1.000 were truncated to 1.000 for presentation.

**Table S4.** Summary of pathway analysis results for HBV-ACLF.

| Pathway Name | Total | Hits | *p* | Impact | Hit metabolites |
| --- | --- | --- | --- | --- | --- |
| Tyrosine metabolism | 42 | 13 | 5.71E-10 | 0.654 | L-Noradrenaline, Homogentisic acid, 3-(4-Hydroxyphenyl)pyruvic acid, 3,4-Dihydroxyphenylacetic acid, 3,4-Dihydroxy-L-phenylalanine, Tyrosine, 4-Hydroxyphenylacetaldehyde, 3-Methoxy-4-hydroxyphenylethyleneglycol, 2-Hydroxy-3-(4-hydroxyphenyl)propenoic acid, Dopamine, 3-Methoxy-4-hydroxymandelic acid, 3,4-Dihydroxyphenylacetaldehyde, 2,5-Dihydroxybenzoic acid |
| Taurine and hypotaurine metabolism | 8 | 4 | 9.98E-05 | 0.829 | Hypotaurine, Cysteine, Taurine, L-Cysteic acid |
| Tryptophan metabolism | 41 | 7 | 5.04E-04 | 0.187 | Serotonin, 5-Hydroxyindoleacetic acid, 5-Hydroxykynurenamine, 3-Hydroxykynurenamine, 5-Hydroxyindoleacetaldehyde, 5-Hydroxykynurenine, 4,8-Dihydroxyquinoline |
| Cysteine and methionine metabolism | 33 | 6 | 9.28E-04 | 0.248 | 5'-Methylthioadenosine, Methionine, S-Adenosyl-L-homocysteine, Dehydroalanine, L-Cysteic acid, Cysteine |
| Arginine biosynthesis | 14 | 4 | 1.21E-03 | 0.441 | Arginine, Citrulline, Glutamic Acid, Aspartic acid |
| Histidine metabolism | 16 | 4 | 2.08E-03 | 0.049 | Glutamic Acid, 3-methyl-Histidine, N-Formimino-L-glutamic acid, Aspartic acid |
| Ubiquinone and other terpenoid-quinone biosynthesis | 19 | 4 | 4.09E-03 | 0.045 | 4-Hydroxybenzoic acid, 3-(4-Hydroxyphenyl)pyruvic acid, Homogentisic acid, Tyrosine |
| Pantothenate and CoA biosynthesis | 20 | 4 | 4.97E-03 | 0.007 | Pantothenic acid, Valine, Aspartic acid, Cysteine |
| beta-Alanine metabolism | 21 | 4 | 5.97E-03 | 0.056 | Aspartic acid, Spermine, beta-Alanyl-Lysine, Spermidine |
| Phenylalanine, tyrosine and tryptophan biosynthesis | 4 | 2 | 7.47E-03 | 0.500 | Tyrosine, 3-(4-Hydroxyphenyl)pyruvic acid |
| One carbon pool by folate | 26 | 4 | 1.30E-02 | 0.102 | Folinic acid, Methionine, S-Adenosyl-L-homocysteine, Cysteine |
| Glutathione metabolism | 28 | 4 | 1.69E-02 | 0.030 | Cysteine, Glutamic Acid, Spermine, Spermidine |
| Lysine degradation | 30 | 4 | 2.14E-02 | 0.137 | Saccharopine, L-Allysine, 5-Hydroxylysine, Pipecolic acid |
| Valine, leucine and isoleucine biosynthesis | 8 | 2 | 3.17E-02 | 0.000 | Leucine, Valine |
| Phenylalanine metabolism | 8 | 2 | 3.17E-02 | 0.000 | 2-Hydroxyphenylacetic acid, Tyrosine |
| Arginine and proline metabolism | 36 | 4 | 3.92E-02 | 0.191 | Arginine, Glutamic Acid, Spermine, Spermidine |
| Glycine, serine and threonine metabolism | 33 | 3 | 1.16E-01 | 0.000 | Aminoacetone, Cysteine, 5-Aminolevulinic acid |
| Glycerophospholipid metabolism | 36 | 3 | 1.41E-01 | 0.080 | Ethanolamine phosphate, Ethanolamine,  sn-Glycero-3-phosphoethanolamine |
| Nitrogen metabolism | 6 | 1 | 2.00E-01 | 0.000 | Glutamic Acid |
| Thiamine metabolism | 7 | 1 | 2.29E-01 | 0.000 | Cysteine |
| Purine metabolism | 70 | 4 | 2.49E-01 | 0.039 | Uric acid, Adenine, Deoxyguanosine, Hypoxanthine |
| Alanine, aspartate and glutamate metabolism | 28 | 2 | 2.72E-01 | 0.421 | Glutamic Acid, Aspartic acid |
| Porphyrin metabolism | 31 | 2 | 3.13E-01 | 0.028 | 5-Aminolevulinic acid, Glutamic Acid |
| Pyrimidine metabolism | 39 | 2 | 4.20E-01 | 0.076 | Uridine, Thymine |
| Butanoate metabolism | 15 | 1 | 4.28E-01 | 0.000 | Glutamic Acid |
| Nicotinate and nicotinamide metabolism | 15 | 1 | 4.28E-01 | 0.000 | Aspartic acid |
| Valine, leucine and isoleucine degradation | 40 | 2 | 4.33E-01 | 0.000 | Leucine, Valine |
| Ether lipid metabolism | 20 | 1 | 5.26E-01 | 0.000 | sn-Glycero-3-phosphoethanolamine |
| Glycosylphosphatidylinositol (GPI)-anchor biosynthesis | 32 | 1 | 6.99E-01 | 0.000 | Ethanolamine phosphate |
| Glyoxylate and dicarboxylate metabolism | 32 | 1 | 6.99E-01 | 0.000 | Glutamic Acid |
| Sphingolipid metabolism | 32 | 1 | 6.99E-01 | 0.006 | Ethanolamine phosphate |
| Primary bile acid biosynthesis | 46 | 1 | 8.23E-01 | 0.008 | Taurine |
